# Phenolic compounds, antioxidant activity and sensory evaluation of sea buckthorn (*Hippophae rhamnoides* L.) leaf tea

**DOI:** 10.1002/fsn3.3155

**Published:** 2022-11-24

**Authors:** Qian He, Kailin Yang, Xinyan Wu, Chunhong Zhang, Chunnian He, Peigen Xiao

**Affiliations:** ^1^ Key Laboratory of Bioactive Substances and Resources Utilisation of Chinese Herbal Medicine, Institute of Medicinal Plant Development, Chinese Academy of Medical Sciences Peking Union Medical College Beijing China; ^2^ Baotou Medical College Baotou China

**Keywords:** antioxidant activity, phenolic compounds, sea buckthorn leaf tea, sensory evaluation

## Abstract

Sea buckthorn leaf tea, an emerging potential functional beverage product, has not yet had appropriate product standards and corresponding quality evaluation methods, and its poor taste directly affects the acceptance of the population, thus limiting its market consumption potential. In this study, two major packaging forms of sea buckthorn leaf tea available in the Chinese market were selected. The contents of total phenolics, total flavonoids, and 10 phenolic compounds, as well as the in vitro antioxidant capacity and sensory characteristics of sea buckthorn leaf tea were analyzed. Results showed that the quality of sea buckthorn leaf tea in the Chinese market varied widely. The total phenolic content, total flavonoid content, antioxidant activity, and consumer acceptance of bagged sea buckthorn leaf tea were higher than those of bulk sea buckthorn leaf tea. Multifactorial statistical analysis showed that the taste astringency of sea buckthorn leaf tea was closely related to ellagic acid and isorhamnetin‐3‐O‐neohesperidin. Furthermore, isorhamnetin‐3‐O‐neohesperidin had a greater effect on the antioxidant activity of sea buckthorn leaf tea. Therefore, ellagic acid and isorhamnetin‐3‐O‐neohesperidin can be used as potential quality markers for sea buckthorn leaf tea. This work provides a reference for taste improvement and quality control of sea buckthorn leaf tea.

## Graphical abstract

In this study, the quality and taste of sea buckthorn leaf tea in the Chinese market were analyzed by the UPLC technique, in vitro antioxidant activity assay, and sensory evaluation. The results showed that ellagic acid was an important factor affecting the bitterness of sea buckthorn leaf tea. Ellagic acid and isorhamnein‐3‐O‐neohesperidin could be used as potential quality markers for buckwheat leaf tea. This work provides a reference for taste improvement and quality control of sea buckthorn leaf tea for the promotion and application of sea buckthorn leaf tea as a potential functional beverage product.

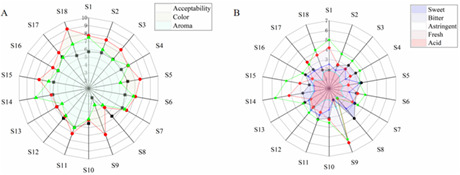

## 1 INTRODUCTION

Sea buckthorn (*Hippophae rhamnoides* L.), belonging to the Elaeagnaceae family, is a thorny deciduous shrub (Büyükokuroǧlu & Gülçin, 2009). Sea buckthorn is widely distributed in many countries, such as China, India, and Mongolia (Morikawa et al., 2015). It is a drought‐tolerant and hardy plant that is used in land reclamation and farmland conservation (Li & Schroeder, 1996). Sea buckthorn berries have been used in traditional medicine for a long history. In the modern food industry, sea buckthorn berries can be processed into fruit powder, beverage, and oil, which have been widely studied and applied (Pundir et al., 2021).

The leaves of sea buckthorn, as another part of sea buckthorn with abundant resources, are not fully utilized. The leaves have attracted increasing attention because of its potential health value. Studies on in vitro and in vivo pharmacological activities have shown that sea buckthorn leaves have antioxidant, antibacterial, antiviral, antitumor, and immunomodulatory effects (Geetha et al., 2003; Jain et al., 2008; Li, Liu, et al., 2021). Population application history investigations and animal studies have shown that sea buckthorn leaves have a good safety profile and no adverse effects in rats (Wang et al., 2017). Sea buckthorn leaves contain a variety of phytochemicals, such as polyphenols, polysaccharides, flavonoids, carotenoids, and saponins (Trivedi & Sati, 2022). In this regard, sea buckthorn leaves are a potential ingredient because of its nutritional and medicated components that are beneficial to human health. As a result, the Chinese government approved the use of sea buckthorn leaves as a common food ingredient in 2013. The development and application of sea buckthorn leaf as a raw material in food, medicine, animal feed, and even cosmetics have rapidly developed (Beveridge et al., 1999; Li, Liu, et al., 2021).

In recent years, scholars have focused on utilizing sea buckthorn leaves and converting them into a potential functional beverage product due to their health benefits. After drying at high temperatures, sea buckthorn leaf tea retains considerable nutritional value and is comparable with commonly consumed vegetables (Tanwar et al., 2018). The common forms of sea buckthorn leaf tea on the market are bulk sea buckthorn leaf tea (unpackaged) and bagged sea buckthorn leaf tea. Sea buckthorn leaf tea contains a variety of functional ingredients with many health benefits, such as lipid lowering, weight loss, and antioxidation (Cho et al., 2014; Lee et al., 2011).

Sea buckthorn leaf tea is an emerging commodity and is available in various forms such as loose sea buckthorn leaf tea and bagged sea buckthorn leaf tea when sold in the market. Since sea buckthorn is widely distributed in China, differences in ecological environment, genetic germplasm, and processing method may significantly affect the chemical composition and activity of sea buckthorn leaf tea (Tanwar et al., 2018). However, no suitable quality evaluation methods and standards are available yet for this product. As a potential functional beverage, the taste of sea buckthorn leaf tea has a direct impact on its market consumption and acceptance. Few reports are available on factors affecting the taste of sea buckthorn leaf tea. Therefore, sea buckthorn leaf tea currently available in the Chinese market should be investigated to establish quality evaluation methods and explore key factors affecting taste. In particular, the correlation between the phenolic composition of sea buckthorn leaves and their taste should be established to expand the utilization of sea buckthorn leaf tea as a potential health resource.

The aim of this study was to investigate the phenolic composition, sensory quality, and antioxidant activity of sea buckthorn leaf tea available in the Chinese market, to investigate the important factors affecting the taste of sea buckthorn leaf tea, and to provide valuable data for the quality evaluation of sea buckthorn leaf tea.

## 2 MATERIALS AND METHODS

### 2.1 Chemicals and materials

The following standards with purity higher than 98% were purchased from Yuanye Biotechnology Co., Ltd.: isoquercitrin, isorhamnetin‐3‐O‐neohesperidoside, narcissin, quercitrin, ellagic acid, gallocatechin, apigenin, rutin, and kaempferol. Epicatechin was obtained from Chengdu Pulse Biotechnology Co., Ltd. Citric acid, sucrose, tannic acid, sodium glutamate, and quinine hydrochloride was acquired from Adamas Beta Chemical Reagents Co., Ltd. 2,2′‐Azino‐*bis*‐(3‐ethylbenzthiazoline‐6‐sulphonate acid; ABTS) and ferric ion‐reducing antioxidant power (FRAP) kits were provided by Shanghai Biyuntian Biotechnology Co., Ltd. Diphenyl‐1‐picrylhydrazyls (DPPH) was supplied by Sigma‐Aldrich. Chromatography‐grade formic acid was obtained from Shanghai Aladdin Reagent Co., Ltd. Methanol was acquired from Tianjin Beilian Fine Chemical Depot. Experimental ultrapure water was produced with a Mili‐Q system (Millipore Corp.).

### 2.2 Sample materials

Sea buckthorn leaf tea samples (No. S1–S18) were purchased in the Chinese market and identified by Prof. Chunnian He, a researcher at the Institute of Medicinal Plant Development in Beijing, China. The source and lot numbers of the samples are shown in Table 1.

**TABLE 1:** Samples of 18 sea buckthorn leaf teas

Number	Manufacturer (China)	Factory time	Type
S1	Xining, Qinghai	June 2020	Sea buckthorn leaf tea bag
S2	Dingxi, Gansu	July 2020	
S3	Zhangye, Gansu	July 2020	
S4	Xinzhou, Shanxi	July 2020	
S5	Lvliang, Shanxi	July 2020	
S6	Da Hinggan Ling Prefecture, Heilongjiang	July 2020	
S7	Xi'an, Shaanxi	January 2021	
S8	Xinjiang	March 2021	Bulk sea buckthorn leaf tea
S9	Tacheng, Xinjiang	June 2021	
S10	Aksu, Xinjiang	January 2021	
S11	Urumqi, Xinjiang	March 2021	
S12	Kashgar, Xinjiang	June 2020	
S13	Jilin City, Jilin Province	July 2020	
S14	Chaoyang, Liaoning	June 2021	
S15	Chaoyang, Liaoning	June 2020	
S16	Zibo, Shandong	June 2020	
S17	Tongliao, Inner Mongolia	June 2021	
S18	Tongliao, Inner Mongolia	June 2021	

### 2.3 Preparation of extracts

In an ultrasonic extractor, about 1 g of tea powder was extracted with 25 ml of water at a temperature of 50°C and a power of 300 W for 40 min. The extracts were stored at 4°C prior to further assay.

### 2.4 Quantitative determination of 10 compound contents by UPLC‐DAD

Quantification of 10 phenolic compounds was performed on a Thermo Ultimate 3000 UPLC system equipped with a DAD‐3000RS (Thermo Fisher Scientific). A Waters ACQUITY UPLC BEH C18 (2.1 mm × 100 mm, 1.7 μm) column was used. The mobile phase consisted of formic acid–water (0.1:100, v/v; A) and acetonitrile (B), and the gradient elution procedure was as follows: 0–10 min, 2%–10.7% B; 10–18 min, 10.7%–10.7% B; 18–25 min, 10.7%–16.3% B; 25–30 min, 16.3%–29.8% B; 30–35 min, 29.8%–45% B; and 35–40 min, 45%–90% B. The flow rate was 0.3 ml/min, and the injection volume was 2 μl. The temperatures of the column and sample tray were 30 and 10°C, respectively. The detection wavelengths were set at 360, 280, and 254 nm. Chromeleon 7 software was used to acquire and analyze data.

A mixture stock standard solution containing 90.53 μg/ml epicatechin, 5.92 μg/ml isorhamnetin‐3‐O‐neohesperidoside, 873.88 μg/ml narcissin, 19.01 μg/ml isoquercitrin, 1500.32 μg/ml ellagic acid, 27.09 μg/ml rutin, 25.60 μg/ml quercitrin, 6.76 μg/ml catechin, 9.47 μg/ml kaempferol, and 6.33 μg/ml apigenin was diluted by 1, 2, 2.5, 3, and 4 times to obtain standard solutions for plotting of standard curves. Prior to UPLC analysis, all solutions were stored at 4°C and filtered through 0.22 μm nylon micropore membranes.

The method was validated for linearity, precision, repeatability, stability, and recovery rates following the International Conference on Harmonization (ICH) guideline (ICH Q2(R1), 2005).

### 2.5 The content of flavonoids and tannins

In order to more fully reflect the content of polyphenols in sea buckthorn leaf tea, a semiquantitative method was temporarily adopted. First, the chromatographic peaks of sea buckthorn leaf tea were identified based on the UV characteristic spectra of flavonoids and tannins (the UV absorption spectra of flavonoids in methanol mainly appear between 300 and 400 nm and 240 and 280 nm; and the tannin has strong characteristic absorption at about 275 nm). Furthermore, the contents of individual flavonoids and tannins were calculated by comparing their peak areas with those of isoquercitrin and ellagic acid standards, respectively. Finally, the contents of all single flavonoid or tannin were added to obtain the total contents of flavonoids and tannins in sea buckthorn leaf tea.

### 2.6 Antioxidant activity assays

In vitro antioxidant activity of ultrasonic extracts of sea buckthorn leaf tea was determined by FRAP, DPPH, and ABTS methods, respectively.

#### 2.6.1 FRAP method

Total antioxidant capacity was evaluated using the FRAP method described by Li, Li, et al. (2021). About 15 μl of sea buckthorn leaf tea solution was placed separately in a 96‐well plate, mixed with 180 μl of FRAP working solution, and shaken thoroughly for 10 s. The solution was incubated for 6 min at 37°C, and absorbance was recorded at 734 nm. The results were expressed as mg TE/g.

#### 2.6.2 DPPH method

DPPH method was used to determine the antioxidant activity with reference to literature (Gülçin et al., 2011; Köksal & Gülçin, 2008). In brief, 0.2 mM DPPH·solution in ethanol was prepared, and 15 μl of sea buckthorn leaf tea solution extract plus 180 μl of 0.2 mM DPPH solution was incubated at 37°C for 30 min under light protection. Absorbance was recorded at 517 nm. The standard curve of Trolox was developed. The antioxidant capacity of the samples was expressed as milligrams of Trolox equivalents per gram of dried sample (mg TE/g).

#### 2.6.3 ABTS method

The ABTS radical scavenging activity of sea buckthorn leaf tea was referenced from the method of Li, Li, et al. (2021). ABTS was blue‐green in color and had a characteristic absorbance at 734 nm. About 10 μl of sea buckthorn leaf tea solution was placed separately in a 96‐well plate, mixed with 200 μl of ABTS + working solution, and shaken thoroughly for 10 s. The sample was incubated at room temperature for 5 min, and absorbance was recorded at 734 nm. The results were expressed as mg TE/g.

#### 2.6.4 Analysis of the antioxidant potency composite (APC) index

The APC index was used to evaluate the overall antioxidant activity of sea buckthorn leaf tea (Peng et al., 2019). APC index was calculated using the following formula: APC index (%) = ([DPPH value]/[the maximum DPPH value] + [ABTS value]/[the maximum ABTS value] + [FRAP value]/[the maximum FRAP value])/3 × 100.

### 2.7 Sensory evaluation

Each sea buckthorn leaf tea (1:50, w/v) was steeped at 80°C for 5 min, and 5 ml of each tea infusion was poured into a disposable plastic cup and cooled to room temperature. Fifteen trained panelists including 11 women and 4 men aged between 20 and 45 years evaluated the product in the laboratory. Each person evaluated 18 sea buckthorn leaf tea samples. The following attributes were evaluated: color, aroma, sour, bitter, sweet, umami, astringent, and overall acceptance. The scoring criteria were based on the 10‐component table method described in a previous work (Liu et al., 2018), as shown in Table S2. Each panelist was asked to wash their taste buds with drinking water between different samples at intervals of 1–2 min. Sensory profiles of the samples were developed based on their average score. Overall acceptance was evaluated based on the taste and color of sea buckthorn leaf tea. The standard references for bitterness, astringency, freshness, and sweetness were quinine hydrochloride, tannin, sodium glutamate, and sucrose, respectively.

### 2.8 Statistical analysis

Standard deviation was calculated for each experiment after three repetitions. One‐way analysis of variance (ANOVA, *p* < .05) with Tukey's HSD and Tamhane's test was used to evaluate the results using SPSS 20.0. Pearson's correlation coefficients were determined using SPSS 20.0.

## 3 RESULTS AND DISSCUSSION

### 3.1 Composition and contents of phenolic compounds

An analytical UPLC‐DAD method was developed for the simultaneous determination of 10 phenolic compounds in sea buckthorn leaf tea. The method was validated by determining linearity, precision, repeatability, stability, and recovery rates. Good linear correlations were obtained for the phenolic compounds using this method with *R* ^2^ > .999. Moreover, the relative standard deviations of the repeatability, precision, stability, and recovery of the method were all below 5.00%, and the recovery was within the range of 97.91%–102.70%. The results confirm the validity of the method for the evaluation of sea buckthorn leaf tea (Table S1). The UPLC‐DAD chromatogram of a representative sample S3 mixed with a standard solution is shown in Figure S1.

The results of ultrasonic‐assisted extraction of sea buckthorn leaf tea are as follows: ten phenolic compounds including two catechins (catechin and epicatechin), one phenolic acid (ellagic acid), one flavonoid (apigenin), and six flavonols (isoquercitrin isorhamnete‐3‐O‐neohesperidin, aquaporin, quercetin, rutin, and kaempferol) were detected from the sea buckthorn leaf tea. Ellagic acid was the major compound in all the test sample (Ciesarová et al., 2020), with the highest content in S1 (Xining, Qinghai, 5.77 mg/g), S2 (Xining, Qinghai, 5.87 mg/g), and S13 (Jilin City, Jilin Province, 4.83 mg/g; Table 2).

**TABLE 2:** The contents of flavonoids and polyphenolic compounds in 18 teas

Number	Isoquercitrin (mg/g)	Ellagic acid (mg/g)	Rutin (mg/g)	Narcissin (mg/g)	Quercitrin (mg/g)	Kaempferol (mg/g)	Catechin (mg/g)	Epicatechin (mg/g)	Isorhamnetin‐3‐O‐neohesperidoside (mg/g)	Apigenin (mg/g)
S1	0.82 ± 0.02^b^	5.77 ± 0.05^a^	0.29 ± 0.01^ef^	0.58 ± 0.01^b^	0.74 ± 0.01^b^	0.056 ± 0.001^fg^	3.38 ± 0.13^a^	0.40 ± 0.02^a^	0.002 ± 0.001^a^	–
S2	0.35 ± 0.03^fg^	5.87 ± 0.25^a^	0.69 ± 0.06^b^	0.27 ± 0.02^d^	1.05 ± 0.05^d^	0.051 ± 0.001^g^	–	0.22 ± 0.01^ef^	0.007 ± 0.001^g^	–
S3	0.35 ± 0.03^fg^	3.32 ± 0.21^f^	0.09 ± 0.01^k^	0.23 ± 0.01^de^	0.25 ± 0.02^de^	0.036 ± 0.001^h^	–	0.33 ± 0.03^b^	0.005 ± 0.001^h^	–
S4	0.26 ± 0.01^j^	1.93 ± 0.06^ij^	0.19 ± 0.02^ghi^	0.11 ± 0.01^fg^	0.39 ± 0.02^fg^	0.032 ± 0.001^h^	–	–	0.002 ± 0.001^j^	–
S5	0.37 ± 0.01^f^	3.33 ± 0.06^f^	0.22 ± 0.01^gh^	0.20 ± 0.01^e^	0.40 ± 0.01^e^	0.069 ± 0.003^d^	–	0.29 ± 0.02^cd^	0.008 ± 0.001^g^	–
S6	0.31 ± 0.02^ghi^	2.86 ± 0.13^g^	0.30 ± 0.01^e^	0.25 ± 0.01^de^	0.50 ± 0.02^de^	0.116 ± 0.004^a^	1.38 ± 0.06^b^	0.25 ± 0.01 ^e^	0.017 ± 0.001^bc^	0.003 ± 0.001^a^
S7	0.74 ± 0.02^c^	2.37 ± 0.19^h^	0.15 ± 0.01^ij^	0.61 ± 0.03^b^	0.30 ± 0.01^b^	0.059 ± 0.002^ef^	–	–	0.012 ± 0.001^ef^	–
S8	0.29 ± 0.0^hij^	1.57 ± 0.02^j^	0.63 ± 0.02^c^	0.22 ± 0.01 ^e^	1.19 ± 0.03 ^e^	0.063 ± 0.003 ^e^	–	0.09 ± 0.01^h^	0.003 ± 0.001^ij^	–
S9	0.43 ± 0.01 ^e^	1.59 ± 0.06^j^	0.87 ± 0.02^a^	0.63 ± 0.03^b^	1.84 ± 0.05^b^	–	1.35 ± 0.15^b^	0.13 ± 0.01^g^	0.013 ± 0.001^de^	–
S10	0.64 ± 0.01^d^	1.69 ± 0.18^j^	0.45 ± 0.02^d^	0.46 ± 0.03^c^	0.63 ± 0.01^c^	0.108 ± 0.001^b^	0.62 ± 0.03^c^	‐	0.014 ± 0.001^d^	–
S11	0.27 ± 0.02^ij^	4.34 ± 0.13^cd^	0.08 ± 0.01^k^	0.11 ± 0.01^fg^	0.36 ± 0.02^fg^	0.035 ± 0.001^h^	–	0.23 ± 0.01^ef^	0.017 ± 0.001^c^	–
S12	0.29 ± 0.02^hij^	2.25 ± 0.03^hi^	0.60 ± 0.03^c^	0.24 ± 0.01^de^	1.11 ± 0.01^de^	0.033 ± 0.001^h^	–	0.25 ± 0.02 ^e^	0.018 ± 0.001^bc^	–
S13	0.31 ± 0.02^hi^	4.83 ± 0.22^b^	0.17 ± 0.01^ij^	0.11 ± 0.01^fg^	0.39 ± 0.01^fg^	0.057 ± 0.005^f^	1.35 ± 0.09^b^	0.25 ± 0.02 ^e^	0.017 ± 0.001^bc^	–
S14	0.21 ± 0.01^k^	3.93 ± 0.18 ^e^	0.15 ± 0.01^ij^	0.07 ± 0.01^g^	0.39 ± 0.01^g^	0.032 ± 0.001^h^	–	0.21 ± 0.01^f^	0.014 ± 0.001^d^	–
S15	0.34 ± 0.01^fgh^	4.07 ± 0.13^de^	0.24 ± 0.02^fg^	0.13 ± 0.01^f^	0.49 ± 0.01^f^	0.072 ± 0.001^d^	–	0.25 ± 0.01^de^	0.019 ± 0.001^ab^	–
S16	0.88 ± 0.04^a^	2.42 ± 0.09^h^	0.18 ± 0.01^hi^	0.94 ± 0.05^a^	0.21 ± 0.01^a^	0.098 ± 0.005^c^	0.68 ± 0.02^c^	0.32 ± 0.01^bc^	0.011 ± 0.001^f^	–
S17	0.30 ± 0.01^hij^	2.38 ± 0.04^h^	0.03 ± 0.01^L^	0.24 ± 0.01^de^	0.17 ± 0.01^de^	0.056 ± 0.002^fg^	–	0.20 ± 0.01^f^	0.004 ± 0.001^hi^	–
S18	0.29 ± 0.01^hij^	4.60 ± 0.16^bc^	0.12 ± 0.01^jk^	0.12 ± 0.01^fg^	0.42 ± 0.01^fg^	–	–	0.21 ± 0.02^f^	0.017 ± 0.001^bc^	0.002 ± 0.001^b^
Sum total	7.45	59.12	5.45	5.52	10.83	0.973	8.76	3.63	0.2	0.007

*Note*: Values are expressed as the mean standard deviation, *n* = 3. Significant differences between different drying methods of each variety (*p* < .05) are marked with a–j.

In this study, on the other hand, quercetin was higher in sea buckthorn leaf tea, which could be due to different processing methods and origins. Samples with higher isorhamnetin derivatives (isorhamnetin and isorhamnete‐3‐O‐neohesperidin) were S15 (Chaoyang, Liaoning) and S11 (Urumqi, Xinjiang). The study reported that the isorhamnetin derivatives content of sea buckthorn leaves was superior to that of quercetin derivatives (Pop et al., 2013). Overall, the highest quantity of nine flavonoids was found in the sea buckthorn leaf tea of S9 (Tacheng, Xinjiang), S8 (Xinjiang), and S1 (Xining, Qinghai).

Among all the test samples, ellagic acid total content was the highest at 59.12 mg/g, consistent with the report that tannin components are mainly found in sea buckthorn leaves (Wang et al., 2021). The study evaluated the sea buckthorn leaf profiles and reported that phenolic acid content is proportional to the flavonoid content (Raudone et al., 2021). However, in the present work, the samples with a more excellent ellagic acid content of sea buckthorn leaf tea all had lower total flavonoid content. This finding could be due to high‐temperature enzyme inactivation, thus better preserving the phenolic acids in the sea buckthorn leaf tea (Ma et al., 2019). The contents of catechins and apigenin were low in the sea buckthorn leaf tea.

**FIGURE 1:**
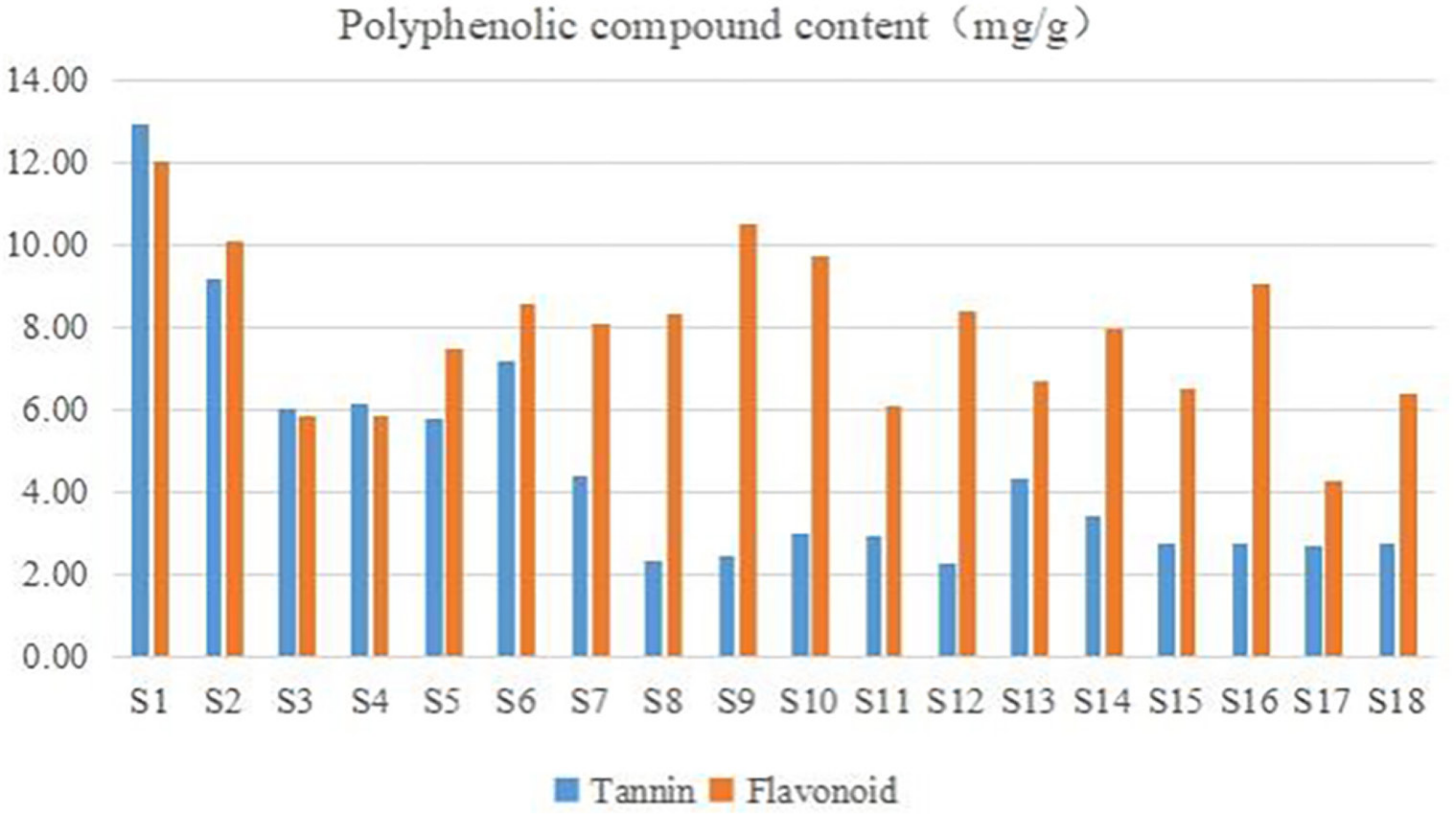
Polyphenols content of sea buckthorn leaf tea

The content of sea buckthorn leaf tea polyphenolic compounds is shown in Figure 1. The content of tannins ranged from 12.93 to 2.28 mg/g and the content of flavonoids ranged from 12.02 to 4.26 mg/g. S1 (Xining, Qinghai, 11.98 mg/g), S2 (Dingxi Gansu, 10.09 mg/g), and S9 (Tacheng, Xinjiang, 10.53 mg/g) had higher flavonoid content. S1 (Xining, Qinghai, 12.93 mg/g), S2 (Dingxi, Gansu, 9.05 mg/g), and S6 (Da Hinggan Ling Prefecture, Heilongjiang, 7.12 mg/g) had higher tannin content. In addition, the flavonoid and tannin contents of S17 (Tongliao, Inner Mongolia) were low. S17 was identified as sea buckthorn leaf black tea. In general, black tea decreases flavonoids and flavonoid glycosides after a specific processing step during fermentation (Feng et al., 2020). The bagged sea buckthorn leaf tea (S1–S7, 7.35 ± 2.85 mg/g) contained higher levels of tannins than the bulk sea buckthorn leaf tea (S8–S18, 3.01 ± 0.59 mg/g; *p* < .01). However, there was no significant difference in total flavonoids content between the two types of tea (*p* > .05). This finding illustrates the higher degree of crushing in bagged tea than in bulk tea. It also illustrates that proper crushing of tea leaves can increase the extraction rate of active ingredients (Danna et al., 2016).

### 3.2 Analysis of antioxidant activity

According to literature reports, evaluation of the antioxidant activities of natural antioxidants is difficult using a single method. Therefore, the processes involved in employing different assay principles are necessary (Higdon & Frei, 2003; Liu et al., 2019; Xu et al., 2017). In the present study, three commonly used different assays such as FRAP (reducing Fe^3+^ to Fe^2+^), ABTS (scavenging ATBS radical), and DPPH (removes DPPH free radicals) were combined to evaluate the antioxidant activities of tea extracts (Table 3).

**TABLE 3:** Antioxidant activity of sea buckthorn leaf tea

Number	ABTS (mmol TE/g)	DPPH (mmol TE/g)	FRAP (mmol TE/g)	APC comprehensive index (%)
S1	5.72 ± 0.34^bc^	50.21 ± 2.71^a^	17.94 ± 0.24^a^	88.48
S2	5.37 ± 0.61^bc^	50.12 ± 1.28^a^	16.72 ± 0.33^bc^	84.80
S3	9.84 ± 0.08 ^a^	45.01 ± 1.47^bc^	16.81 ± 0.98^bc^	96.73
S4	6.75 ± 0.68^b^	42.04 ± 0.83^bcdef^	14.08 ± 0.34^g^	78.85
S5	9.58 ± 0.25^a^	42.63 ± 1.14^bcde^	14.51 ± 0.22^efg^	89.68
S6	0.81 ± 0.55^h^	42.78 ± 0.38^bcde^	13.95 ± 0.31^g^	58.96
S7	1.42 ± 0.33^gh^	44.48 ± 1.77^bcd^	16.18 ± 0.30^cd^	66.60
S8	4.91 ± 0.37^cd^	45.64 ± 1.31^bc^	17.77 ± 0.50^a^	82.36
S9	2.91 ± 0.78^efg^	40.26 ± 1.12^def^	13.95 ± 0.15^g^	64.40
S10	5.98 ± 0.62^bc^	46.22 ± 4.25^bc^	17.34 ± 0.56^ab^	85.51
S11	2.06 ± 0.86^fgh^	44.69 ± 0.52^bcd^	14.84 ± 0.12^efg^	66.23
S12	2.38 ± 0.79^efgh^	43.20 ± 0.69^bcde^	15.16 ± 0.39^ef^	66.97
S13	2.30 ± 0.72^efgh^	41.36 ± 0.99^cdef^	15.27 ± 0.35^def^	65.69
S14	1.44 ± 0.33^gh^	41.97 ± 0.19^bcdef^	15.13 ± 0.23^ef^	62.90
S15	3.29 ± 0.25^ef^	38.19 ± 0.18^fg^	14.42 ± 0.19^fg^	65.25
S16	1.24 ± 0.43^h^	20.69 ± 0.61^h^	12.00 ± 0.04^h^	41.86
S17	6.01 ± 0.66^bc^	39.04 ± 1.68^efg^	15.54 ± 0.13^de^	77.26
S18	3.82 ± 0.73^de^	35.74 ± 1.17^g^	14.10 ± 0.22^g^	64.78

*Note*: Values are expressed as the mean standard deviation, *n* = 3. Significant differences between different drying methods of each variety (*p* < .05) are marked with a–h.

The FRAP values were 12.00 ± 0.04 to 17.94 ± 0.24 mmol TE/g for different tea beverages. The ABTS values varied widely among the different tea beverages, with the maximum value being 12 times higher than the minimum value. The DPPH values varied widely among the different tea beverages with a minimum value of 20.69 ± 0.61 and the maximum value of 50.21 ± 2.71 mmol TE/g. The results showed that sea buckthorn leaf tea had higher ability to scavenge DPPH free radicals than the two other modalities. The difference in correlation coefficients between the three different antioxidant methods may be due to the other measurement principles of different evaluation methods.

Antioxidant potency composite index was calculated to comprehensively compare the antioxidant activity from sea buckthorn leaf tea (Table 3). The ranked antioxidant activity from the largest to the smallest was as follows: S3 (Zhangye, Gansu) > S5 (Lvliang, Shanxi) > S1 (Xining, Qinghai) > S10 (Aksu, Xinjiang) > S2 (Dingxi, Gansu) > S8 (Xinjiang) > S4 (Xinzhou, Shanxi) > S17 (Tongliao, Inner Mongolia) > S12 (Kashgar, Xinjiang) > S7 (Xi'an, Shaanxi) > S11 (Urumqi, Xinjiang) > S13 (Jilin City, Jilin Province) > S15 (Chaoyang, Liaoning) > S18 (Tongliao, Inner Mongolia) > S9 (Tacheng, Xinjiang) > S14 (Chaoyang, Liaoning) > S6 (Da Hinggan Ling Prefecture, Heilongjiang) > S16 (Zibo, Shandong). The difference between the maximum and minimum values of the APC index is 33.83%. This finding was closely related to the content of flavonoids and phenolic acids in sea buckthorn leaf tea. Phenolic compounds, such as flavonoids, phenolic acids, and tannins, are known to be the major providers of antioxidant capacity in plants (Yang et al., 2019).

The average APC index of the bagged sea buckthorn leaf tea (S1–S7, 80.59 ± 13.48) was found to be stronger than that of the bulk sea buckthorn leaf tea (S8–S18, 67.56 ± 11.60; *p* < .05). This result highlighted that the antioxidant capacity of the bagged sea buckthorn leaf tea was stronger than that of the loose sea buckthorn leaf tea (*p* < .05). We speculate that this might be due to the higher degree of grinding tea leaves for bagged tea than that for loose sea buckthorn leaf tea; the degree of grind is positively correlated with antioxidant capacity (Q. Xu et al., 2021).

### 3.3 Sensory evaluation

**FIGURE 2:**
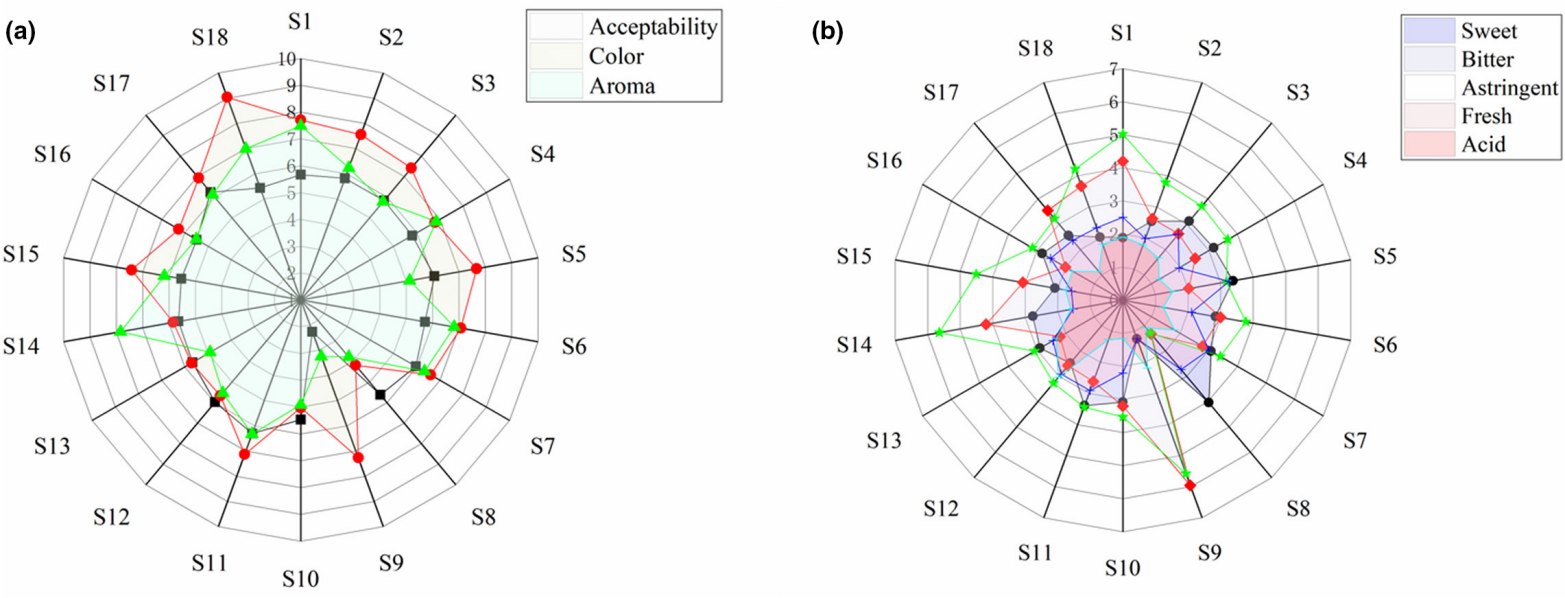
Sea buckthorn leaf tea taste evaluation chart

Taste is one of the important quality factors that influence consumer preferences after variety (Yu et al., 2020). Therefore, sensory analysis of sea buckthorn leaf tea was carried out. The experiment is illustrated in Figure 2. Color is an important quality criterion that influences consumer preferences because it is the first attribute perceived by consumers (Lima et al., 2019). The results showed that sea buckthorn leaf tea infusion was yellow‐brown, and the color difference was not significant (*p* > .05). The common explanation for the yellowish‐brown color of tea infusion is that it is determined by water‐soluble flavonoids such as kaempferol, isoquercitrin, and rutin. The result showed no significant difference in the aroma evaluation of the sea buckthorn leaf teas.

Flavor is one of the important factors of acceptance among consumers. Astringency was found in all tea infusion from 1.29 ± 1.39 to 5.64 ± 1.62. The sweet scores pattern showed the highest score at S18 (Tongliao, Inner Mongolia, 3.38), followed by S4 (Xinzhou, Shanxi, 1.24). Acid flavor was found in all tea infusion from 1.09 ± 1.63 to 3.04 ± 2.36. The fresh scores pattern showed the highest score at S18 (Tongliao, Inner Mongolia, 3.26), followed by S4 (Xinzhou, Shanxi, 1.24). Bitterness was found in all tea infusion from 2.00 ± 1.53 to 5.96 ± 2.23. Based on the score of the sensory characteristics, the corresponding radar chart was created. Statistical differences were found in bitterness, astringency, and acid in sea buckthorn leaf tea samples (*p* < .05). Ma et al. (2019) claimed that the astringency perception threshold of sea buckthorn leaves is low. However, their results were inferred from this fact. This fact is that sea buckthorn leaves are rich in flavonol glycosides, which have little effect on astringency (Scharbert & Hofmann, 2005). Therefore, our experimental results on the predominantly bitter and astringent taste of sea buckthorn leaf tea are more reliable. It is currently reported that the astringent substances of tea are mainly polyphenols, and the primary phenolic acids in tea are ellagic acid, chlorogenic acid, etc. (Ceci et al., 2018). At the same time, flavonoids are one of the important factors affecting the bitter taste of tea (Zhang et al., 2020). At present, technologies to improve the bitter taste of tea mainly include enzyme treatment, microcapsule technology, food irradiation, and other technologies (Ye et al., 2022). Therefore, these methods can be used to improve the taste of sea buckthorn leaf tea.

Acceptability was found in all 18 tea infusion from 2.27 ± 1.62 to 6.24 ± 1.56. The top three teas with the acceptability were S11 (Urumqi, Xinjiang, 6.36), S17 (Tongliao, Inner Mongolia, 6.03), and S5 (Lvliang, Shanxi, 5.67). S8 (Xinjiang, 2.15) has the lowest acceptance. According to Figure 2, it can be shown that the bitterness and astringency of sea buckthorn leaf tea are closely related to the overall acceptability. Meanwhile, the mean value of acceptability of the bagged sea buckthorn leaf tea (S1–S7) was 5.8 and the mean value of acceptability of the bulk sea buckthorn leaf tea (S8–S10) was 5.4. Hence, bagged tea is more popular among consumers. In summary, the results showed the influence of taste and packaging form on the acceptance of sea buckthorn leaf tea.

### 3.4 Correlation analysis

To explore the relationship between antioxidant activity, sensory evaluation, and individual phenols and flavonoids, we calculated the Pearson's correlation coefficient (Tables 4 and 5). This is because polyphenolic compounds are an important factor affecting the antioxidant properties and taste of sea buckthorn leaf tea (Barbe et al., 2019; Delius et al., 2017).

**TABLE 4:** Correlations between antioxidant activity and main compounds

	ABTS	DPPH	FRAP
ABTS	1	0.34	0.403
DPPH	0.34	1	0.801**
FRAP	0.403	0.801**	1
Tannins	0.321	0.505*	0.384
Flavonoids	−0.176	0.224	0.256
Isoquercitrin	−0.092	−0.23	0.08
Ellagic acid	0.008	0.257	0.171
Rutin	−0.062	0.28	0.205
Narcissin	−0.196	−0.374	−0.11
Quercitrin	−0.084	0.297	0.19
Kaempferol	−0.079	−0.111	0.041
Catechin	−0.117	0.149	0.172
Epicatechin	0.073	−0.16	−0.123
Isorhamnetin‐3‐O‐neohesperidoside	−0.573*	−0.025	−0.139
Apigenin	−0.286	−0.107	−0.298

*Note*: Pearson's correlation at **p < .01, *p < .05. The larger the correlation coefficient is, the redder the cell color is; the smaller the correlation coefficient, the greener the color.

**TABLE 5:** Correlations between sensory evaluation and main compounds

	Sweet	Bitter	Astringent	Fresh	Acid
Sweet	1	−0.818**	−0.767**	0.582**	−0.505*
Bitter	−0.818**	1	0.861**	−0.651*	0.182
Astringent	−0.767**	0.861	1	−0.669**	0.249
Fresh	0.582*	−0.651**	−0.669**	1	0.023
Acid	−0.505*	0.182	0.249	0.023	1
Tannins	−0.127	0.057	0.235	0.05	−0.021
Flavonoids	−0.337	0.315	0.293	−0.162	0.266
Isoquercitrin	−0.153	0.057	0.051	0.2	0.072
Ellagic acid	−0.253	0.048	0.321	−0.014	0.19
Rutin	−0.268	0.227	0.03	−0.302	0.311
Narcissin	−0.263	0.188	0.074	0.043	0.142
Quercitrin	−0.384	0.388	0.149	−0.335	0.361
Kaempferol	0.38	−0.439*	−0.371	0.183	−0.374
Catechin	−0.439*	0.399	0.352	−0.131	0.19
Epicatechin	−0.264	−0.001	0.2	0.154	0.316
Isorhamnetin‐3‐O‐neohesperidoside	−0.484*	0.338	0.45	−0.094	0.473*
Apigenin	−0.126	0.088	0.073	−0.095	−0.16

*Note*: Pearson's correlation at **p < .01, *p < .05. The larger the correlation coefficient is, the redder the cell color is; the smaller the correlation coefficient, the greener the color.

As shown in Table 4, the results indicated that the stronger antioxidant capacity of sea buckthorn leaf tea samples may be due to the strong correlation between antioxidant values with tannins. The Pearson's correlation coefficients between total flavonoid content and antioxidant values were all lower than the Pearson's correlation coefficients between tannin content and antioxidant values. For individual compounds, isorhamnetin‐3‐O‐neohesperidoside has a certain correlation with antioxidant activity. In contrast to earlier findings, we find that ellagic acid was less correlated with antioxidant. We believe that the antioxidant activity of sea buckthorn leaf tea is influenced by species factors. For example, different tea steeping temperatures may all be responsible for the antioxidant activity of the compounds being affected (Pérez‐Burillo et al., 2018).

As shown in Table 5, polyphenolic compounds were positively correlated with bitterness, astringency, and arithmetic and negatively correlated with sweet and fresh. Importantly, ellagic acid and isorhamnetin‐3‐O‐neohesperidoside have correlation with astringent. Most of the available reported studies suggest that the astringency of tea liquids is caused by whole saliva or the interaction of proline‐rich proteins (PRPs) with polyphenols (Georgiades et al., 2014). Meanwhile, catechin and isorhamnetin‐3‐O‐neohesperidin showed moderate correlation with bitterness. This is consistent with the current report that the catechins are important in affecting the bitter taste of the tea infusion (J. Li et al., 2017).

## 4 CONCLUSION

In this study, sea buckthorn leaf teas circulating in the China market were analyzed by UPLC, sensory evaluation, and in vitro antioxidant assay. The results indicate that the quality of sea buckthorn leaf tea varies greatly, and the polyphenol content, antioxidant activity, and sensory evaluation of bagged sea buckthorn leaf tea were better than those of bulk sea buckthorn leaf tea. Importantly, the taste astringency of sea buckthorn leaf tea was closely related to ellagic acid and isorhamnetin‐3‐O‐neohesperidin. Meanwhile, isorhamnetin‐3‐O‐neohesperidin had a greater effect on the antioxidant activity of sea buckthorn leaf tea. Therefore, ellagic acid and isorhamnetin‐3‐O‐neohesperidin are recommended as quality control indicators for sea buckthorn leaf tea. The study provides a reference for the taste improvement of sea buckthorn leaf tea and valuable data for the quality control of sea buckthorn leaf tea. It is beneficial for promoting and applying sea buckthorn leaf tea as potential functional beverage products.

## FUNDING INFORMATION

This research was funded by the Major Project of Strategic Research and Consultation of the Chinese Academy of Engineering (2021‐XZ‐10), the National Key Research and Development program (2021YFE0190100), the CAMS Innovation Fund for Medical Sciences (CIFMS ID: 2021‐I2M‐1‐071), and the Innovation Team and Talents Cultivation Program of National Administration of Traditional Chinese Medicine (ZYYCXTD‐D‐202005).

## CONFLICT OF INTEREST

All authors disclosed no relevant relationships.

## ETHICS STATEMENT

This article does not contain any studies performed with human participants or animals by any of the authors.

## CONSENT TO PARTICIPATE

Corresponding and all the coauthors are willing to participate in this manuscript.

## CONSENT FOR PUBLICATION

All authors are willing for publication of this manuscript.

## Supporting information


Table S1–S2
Figure S1

## Data Availability

Even though adequate data have been given in the form of tables and figures, all authors declare that if more data are required, then the data will be provided on request basis.
