# Supplementary material for: Phenolic compounds, antioxidant activity and sensory evaluation of sea buckthorn (Hippophae rhamnoides L.) leaf tea
Source: Food Sci Nutr. 2022 Nov 24;11(3):1212–22. doi: 10.1002/fsn3.3155 (PMC10003008; doi:10.1002/fsn3.3155)
Supplement: Supplementary file 1 — Table S1–S2 Figure S1 [file FSN3-11-1212-s001.docx]

**Phenolic compounds, antioxidant activity and sensory evaluation of sea buckthorn (*Hippophae rhamnoides* L.) leaf tea**

**Qian He^1,2,3^, Kailin Yang^1,2^, Xinyan Wu^1,2^, Chunhong zhang^3^, Chunnian He^1,2*^, and Peigen Xiao^1,2^**

*^1^Key Laboratory of Bioactive Substances and Resources Utilization of Chinese Herbal Medicine,* *Institute of Medicinal Plant Development, Chinese Academy of Medical Sciences, Peking Union Medical College, Beijing, 100193, China*

*^2^ Laboratory of Bioactive Substances and Resources Utilization of Chinese Herbal Medicine, Ministry of Education, Beijing 100193, China*

*^3^ Baotou Medical College, Baotou , Inner Mongolia,014060, China*

*Corresponding author(s). E-mail(s): *cnhe@implad.ac.cn*;

Contributing authors: heqian971003@163.com

Table S1. (A) Methodological investigation

Table S1. (A) Linear relationship, correlation coefficient and linear range of 10 characteristic components

| Compound | Regression equation | r | Linear range（µg/mL） | LOD  （µg/mL） | LOQ（µg/mL） |
| --- | --- | --- | --- | --- | --- |
| Quercitrin | y=11.809x+0.8402 | 0.9991 | 6.65-25.60 | 0.2376 | 0.4762 |
| Narcissin | y=9.8169x-1.7866 | 0.9994 | 217.13-873.88 | 0.3656 | 1.4350 |
| Isoquercitrin | y=13.315x-0.4443 | 0.9996 | 4.59-19.01 | 0.1223 | 0.3380 |
| Ellagic acid | y=24.955x+13.671 | 0.9990 | 400.52-1500.32 | 0.3260 | 2.3260 |
| Catechin | y=43.974x-0.3067 | 0.9990 | 1.67-6.76 | 0.1025 | 0.4560 |
| Apigenin | y=10.41x+0.4601 | 0.9992 | 1.41-6.33 | 0.1182 | 0.4723 |
| Rutin | y=18.522x-0.1678 | 0.9990 | 6.80-27.09 | 0.3092 | 1.2360 |
| Kaempferol | Y=7.0294x+0.5418 | 0.9999 | 2.42-9.47 | 0.1005 | 0.3603 |
| Isorhamnetin-3-O-neohesperidoside | y=1.0971x-0.0289 | 0.9990 | 1.46-5.92 | 0.1073 | 0.3285 |
| Epicatechin | y=19.178x-2.708 | 0.9992 | 23.61-90.53 | 0.3265 | 1.2350 |

Table S1. (B) The validation results of UPLC (n=6)

| Compound | Precision | | Stability | | Repeatability | | Standard recovery rate (%) | Recovery RSD(%), |
| --- | --- | --- | --- | --- | --- | --- | --- | --- |
|  | tR（RSD%） | Area（RSD%） | tR（RSD%） | Area（RSD%） | tR（RSD%） | Area（RSD%） |  |  |
| Isoquercitrin | 1.81 | 1.47 | 3.40 | 2.41 | 2.74 | 4.34 | 102.70 | 1.52 |
| Ellagic acid | 3.49 | 1.62 | 2.71 | 1.92 | 4.63 | 3.67 | 100.36 | 1.23 |
| Rutin | 2.42 | 2.76 | 2.84 | 3.01 | 5.45 | 2.67 | 101.32 | 0.93 |
| Narcissin | 2.44 | 1.43 | 3.76 | 1.94 | 4.38 | 4.03 | 98.29 | 0.34 |
| Quercitrin | 2.87 | 3.95 | 3.57 | 2.18 | 3.28 | 4.34 | 101.44 | 1.85 |
| Kaempferol | 1.57 | 3.31 | 4.19 | 3.27 | 3.18 | 1.91 | 98.35 | 1.15 |
| Catechin | 1.72 | 1.83 | 0.81 | 0.98 | 4.79 | 2.13 | 97.91 | 1.24 |
| Epicatechin | 0.65 | 0.52 | 0.53 | 0.95 | 3.60 | 0.47 | 100.25 | 0.95 |
| Isorhamnetin-3-O-neohesperidoside | 0.62 | 2.09 | 4.63 | 4.89 | 3.26 | 4.36 | 100.29 | 0.94 |
| Apigenin | 3.29 | 3.38 | 2.52 | 2.97 | 1.60 | 4.46 | 100.46 | 1.36 |

Table S2. The grading standard of Sea buckthorn Leaf Tea

|  | Category | Scoring criteria | Score |
| --- | --- | --- | --- |
| Appearance | Color | Colorless | 0—2 |
|  |  | Light yellow | 2—4 |
|  |  | Yellow | 4—6 |
|  |  | Orange | 6—8 |
|  |  | Orange-Red | 8—10 |
|  | Aroma | No aroma | 0—2 |
|  |  | Weaker aroma | 2—4 |
|  |  | Insignificant tea aroma | 4—6 |
|  |  | Tea aroma is obvious and short-lived | 6—8 |
|  |  | Tea aroma is obvious and long lasting | 8—10 |
| Taste | Sweet | Very sweet | 8—10 |
|  |  | Sweeter | 6—8 |
|  |  | Medium sweet | 4—6 |
|  |  | Not significantly sweet | 2—4 |
|  |  | No sweetness | 0—2 |
|  | Bitter | Very bitter | 8—10 |
|  |  | Bitter | 6—8 |
|  |  | Medium bitter | 4—6 |
|  |  | Not significantly bitter | 2—4 |
|  |  | No bitter | 0—2 |
|  | Astringent | Very astringent | 8—10 |
|  |  | Astringent | 6—8 |
|  |  | Medium astringent | 4—6 |
|  |  | Not significantly astringent | 2—4 |
|  |  | No astringent | 0—2 |
|  | Fresh | Very fresh | 8—10 |
|  |  | Fresh | 6—8 |
|  |  | Medium fresh | 4—6 |
|  |  | Not significantly fresh | 2—4 |
|  |  | No fresh | 0—2 |
|  | Acid | Very acid | 8—10 |
|  |  | Acid | 6—8 |
|  |  | Medium acid | 4—6 |
|  |  | Not significantly acid | 2—4 |
|  |  | No acid | 0—2 |
|  | Acceptance | Very willing to accept | 8—10 |
|  |  | Happy to accept | 6—8 |
|  |  | Acceptable | 4—6 |
|  |  | Not significantly acceptable | 2—4 |
|  |  | Unacceptable | 0—2 |


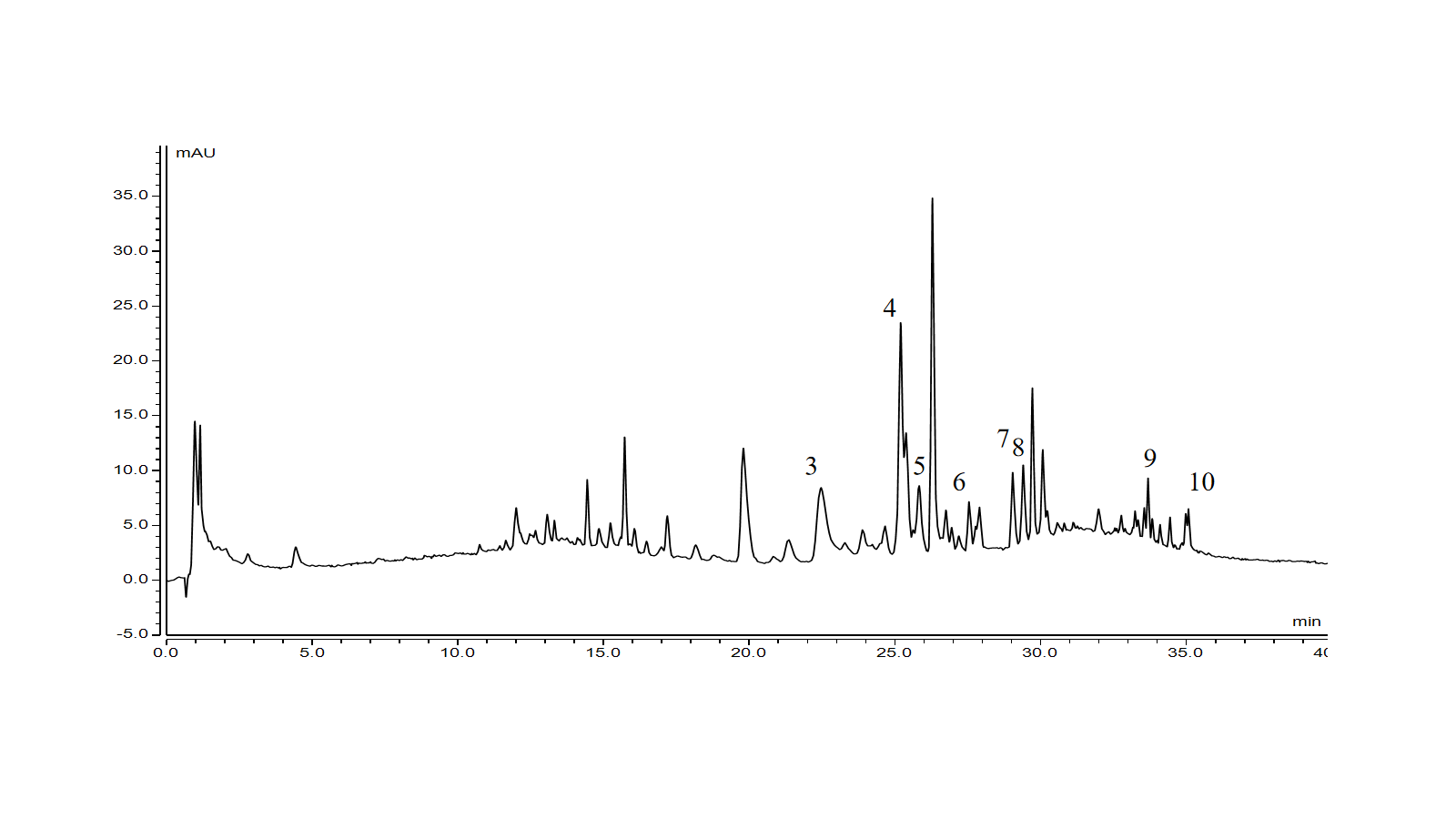


**Figure S1.** UPLC-DAD chromatogram: (A) S3 sample at 360 nm


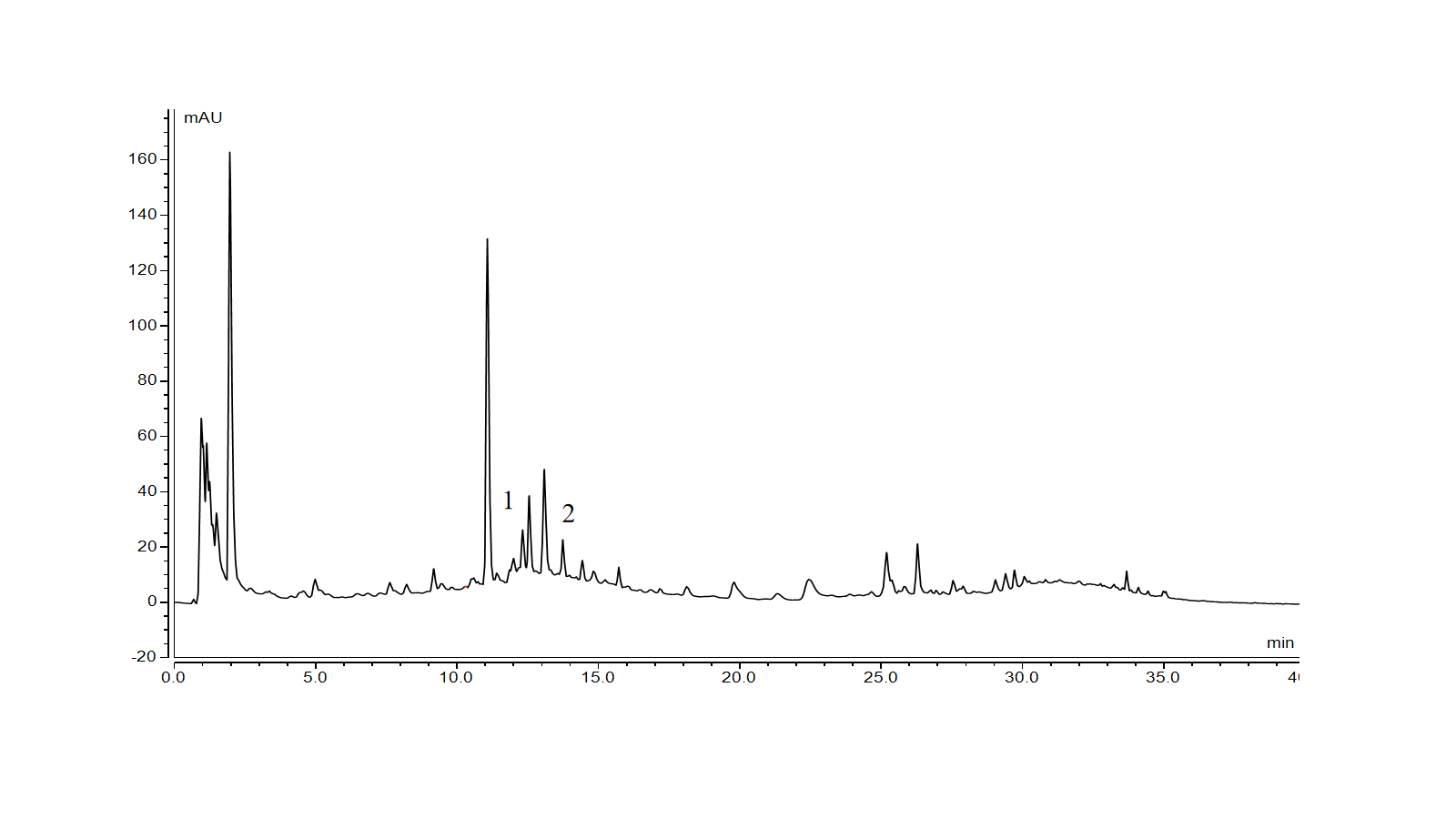


**Figure S1.** UPLC-DAD chromatogram: (B) S3 sample at 280 nm


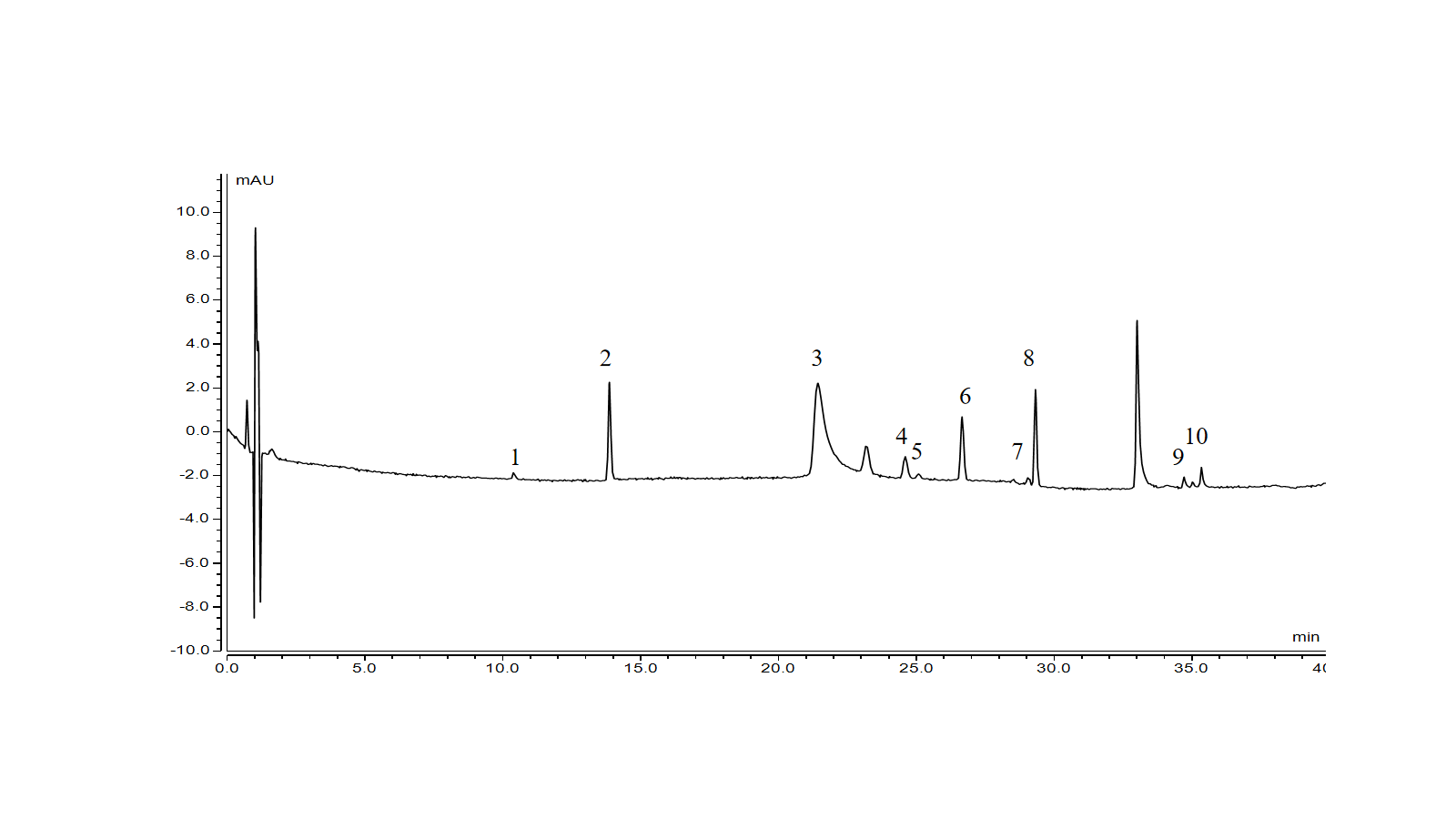


**Figure S1.** UPLC-DAD chromatogram: (C) mixed standard solutions at 254nm

[Compound](javascript:;) 1: Catechin, 2: Epicatechin, 3: Ellagic acid, 4: Isoquercitrin, 5:Rutin, 6: Isorhamnetin-3-O-neohesperidoside, 7: Quercitrin, 8: Narcissin, 9: Apigenin, 10: Kaempferol.
